# Rationale and design of the Sodium Lowering In Dialysate (SoLID) trial: a randomised controlled trial of low versus standard dialysate sodium concentration during hemodialysis for regression of left ventricular mass

**DOI:** 10.1186/1471-2369-14-149

**Published:** 2013-07-15

**Authors:** Joanna Leigh Dunlop, Alain Charles Vandal, Janak Rashme de Zoysa, Ruvin Sampath Gabriel, Imad Adbi Haloob, Christopher John Hood, Philip James Matheson, David Owen Ross McGregor, Kannaiyan Samuel Rabindranath, David John Semple, Mark Roger Marshall

**Affiliations:** 1 South Auckland Clinical School, Faculty of Medical and Health Sciences, University of Auckland, Private Bag 93311. Otahuhu, Auckland 1640, New Zealand; 2 Department of Renal Medicine, Middlemore Hospital, Counties Manukau District Health Board, Private Bag 93311, Otahuhu, Auckland 1640, New Zealand; 3 Faculty of Health and Environmental Sciences, Auckland University of Technology, North Shore Campus, Private Bag 92006, Auckland 1142, New Zealand; 4 Renal Service, North Shore Hospital, Waitemata District Health Board, Private Bag 93503, Takapuna, Auckland 0740, New Zealand; 5 Department of Cardiology, Middlemore Hospital, Counties Manukau District Health Board, Private Bag 93311, Otahuhu, Auckland 1640, New Zealand; 6 Department of Renal Medicine, Auckland City Hospital, Auckland District Health Board, Private Bag 92024, Auckland 0740, New Zealand; 7 Department of Nephrology, Wellington Hospital, Capital & Coast District Health Board, Private Bag 7902, Wellington South, New Zealand; 8 Department of Nephrology, Christchurch Hospital, Canterbury District Health Board, Private Bag 4710, Christchurch, New Zealand; 9 Department of Renal Medicine, Waikato Hospital, Waikato District Health Board, Private Bag 3200, Hamilton 3240, New Zealand

**Keywords:** Home hemodialysis, Dialysis, Left ventricular mass, Sodium, Blood pressure, Fluid overload, Dialysate

## Abstract

**Background:**

The current literature recognises that left ventricular hypertrophy makes a key contribution to the high rate of premature cardiovascular mortality in dialysis patients. Determining how we might intervene to ameliorate left ventricular hypertrophy in dialysis populations has become a research priority. Reducing sodium exposure through lower dialysate sodium may be a promising intervention in this regard. However there is clinical equipoise around this intervention because the benefit has not yet been demonstrated in a robust prospective clinical trial, and several observational studies have suggested sodium lowering interventions may be deleterious in some dialysis patients.

**Methods/design:**

The Sodium Lowering in Dialysate (SoLID) study is funded by the Health Research Council of New Zealand. It is a multi-centre, prospective, randomised, single-blind (outcomes assessor), controlled parallel assignment 3-year clinical trial. The SoLID study is designed to study what impact low dialysate sodium has upon cardiovascular risk in dialysis patients. The study intends to enrol 118 home hemodialysis patients from 6 sites in New Zealand over 24 months and follow up each participant over 12 months. Key exclusion criteria are: patients who dialyse more frequently than 3.5 times per week, pre-dialysis serum sodium of <135 mM, and maintenance hemodiafiltration. In addition, some medical conditions, treatments or participation in other dialysis trials, which contraindicate the SoLID study intervention or confound its effects, will be exclusion criteria. The intervention and control groups will be dialysed using dialysate sodium 135 mM and 140 mM respectively, for 12 months. The primary outcome measure is left ventricular mass index, as measured by cardiac magnetic resonance imaging, after 12 months of intervention. Eleven or more secondary outcomes will be studied in an attempt to better understand the physiologic and clinical mechanisms by which lower dialysate sodium alters the primary end point.

**Discussion:**

The SoLID study is designed to clarify the effect of low dialysate sodium upon the cardiovascular outcomes of dialysis patients. The study results will provide much needed information about the efficacy of a cost effective, economically sustainable solution to a condition which is curtailing the lives of so many dialysis patients.

**Trial registration:**

Australian and New Zealand Clinical Trials Registry number: ACTRN12611000975998

## Background

**Figure 1:**
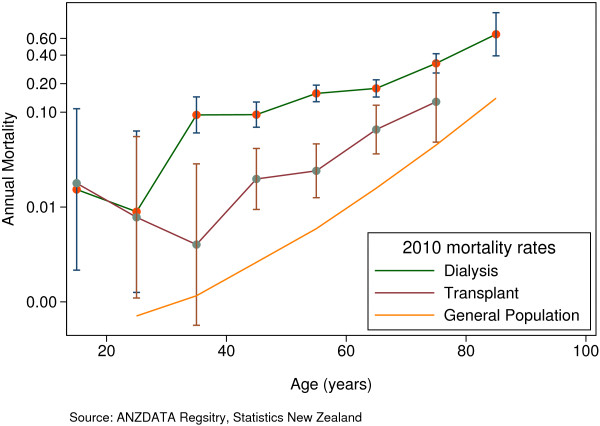
Mortality in New Zealand patients treated by dialysis and transplantation during 2010 compared to the New Zealand general population.

Dialysis is the world’s most utilized modality of renal replacement therapy [1], and enables patients with end stage kidney disease (ESKD) to avoid imminently fatal complications such as hyperkalemia, acidosis, and pulmonary edema and thereby live longer. However, it is apparent that those on dialysis continue to have a uremic toxicity as manifested by a high rate of premature mortality. As is the case in other countries, the median survival of dialysis patients in New Zealand is approximately 4 years, with an overall mortality rate several-fold higher than that of the general population (Figure 1) [2]. This situation is largely attributable to premature cardiovascular (CV) death, with infection playing the next most important role. Although not commonly appreciated, the patients with the highest proportion of CV deaths are those on home hemodialysis (HD), probably as a result of a lower competing risk of infectious death due to patient selection and reduced exposure to nosocomial pathogens. In Australia and New Zealand, 67% of patients on home HD die from CV disease [3].

The mechanism of CV death in dialysis patients appears to be different from that in the general population. Sudden cardiac death (SCD) due to lethal arrhythmia accounts for approximately half of CV deaths in the general population, and is most often a manifestation of coronary heart disease [4]. In contrast, SCD accounts for the majority of CV deaths in dialysis patients and is probably less likely due to sudden coronary ischemia [5,6]. More likely, arrhythmogenesis is triggered by re-entry pathways that are superimposed upon the usual ventricular conducting system by inter-myocardial cell fibrosis [7-9]. This fibrosis results from a synergy between left ventricular (LV) hypertrophy and uremia per se [10,11], and also contributes to a stiff and impaired myocardium and ultimately congestive heart failure [12-16].

There is a range of clinical evidence that supports the crucial role of for LV hypertrophy in SCD among dialysis patients. In a multitude of studies, the presence of LV hypertrophy is a strong independent mortality risk. In a landmark study, LV hypertrophy was associated with a relative risk for death of 2.9, even when adjusted for age, known coronary artery disease, diabetes, and blood pressure (BP) [17]. Of equal importance, recent studies have demonstrated that that the effect of lipid lowering therapy is attenuated in patients with kidney disease, reinforcing the lesser role of sudden rupture of lipid rich plaques as a mechanism of SCD in this group [18-21]. On balance, LV hypertrophy is regarded as a prime and causal risk factor for SCD, and the regression of LV hypertrophy accepted as a validated surrogate primary end point for interventions aiming to reduce CV mortality in dialysis patients [12,22,23].

Persistently elevated blood pressure (BP) and extra-cellular fluid (ECF) overload due to positive salt and water balance are significant contributors to on-going LV hypertrophy with conventional (non-extended-hours) dialysis [24-33]. Current methods to control these factors with either drug therapy or ultra-filtration in patients on conventional dialysis can be effective but are often inadequate in routine clinical practice [27,34-38]. The most effective intervention to improve LV hypertrophy is extended-hours or frequent HD. A randomised controlled trial of extended-hours HD demonstrated a 7.7% reduction in LV mass over a 6month period for patients dialysed in this manner, as opposed to a stable LV mass in those dialysed conventionally [35]. Similar findings were published in the Frequent Haemodialysis Network trial [34]. In both studies, regression of LV mass was associated with improved measures of BP and ECF volume control. Several observational studies have reported similar findings [39,40].

Not all patients, however, are able to manage extended-hours or frequent HD and not all health systems are able to deliver such programs. A more accessible alternative may be available in reducing sodium exposure through lower dialysate [Na+]. Sodium loading by either excessive dietary intake or excessive diffusion via dialysate has been shown to increase both BP and intra-dialytic weight gain (IDWG) [41]. Moreover, elevation in plasma [Na+] can induce hypertension independently of EC fluid volume, through mechanisms that probably include stiffening of vascular endothelium [42-46]. A number of observational studies as well as small and often uncontrolled clinical studies have shown that lower dialysate [Na+] associates with less thirst [47-55], lower IDWG [48,49,51-53,56-78], lower ECF volume [66,76,79,80], and lower BP [48,51,54,56,61-69,75,81-83], with only a minority of studies being completely negative [47,77,84-88]. A typical example can be found in preliminary research by the SoLID trial research team, who previously showed that a decrease in dialysate [Na+] by 3 mM in 52 facility based patients was well tolerated and reduced systolic and diastolic BP by 4–5 and 2–3 mmHg, respectively [89]. Improvement in intermediary outcomes such as BP suggest that lower dialysate [Na+] could be beneficial for improving LV hypertrophy as well. There have been only two studies examining the effect of lower dialysate [Na+] on LV structure and function [90,91]. One study reported an associated decrease in LV volumes, although both were too brief to assess for changes in LV mass.

However, the potential benefits of lower dialysate [Na+] should be weighed against a potential “dark side”. Several large and well performed observational analyses have shown an association between lower dialysate [Na+] and higher mortality risk, notably in those patients with a “frail” phenotype characterized by low serum [Na+], diabetes mellitus, coronary artery disease, CV disease, congestive heart failure, cerebrovascular disease, lung disease, and cancer [72,92,93]. The most plausible explanation for these observations relates to decreased hemodynamic stability with lower dialysate [Na+], and the vulnerability of “frail” patients to intra-dialytic hypotension, an unquestionably threatening condition associated with myocardial stunning and all-cause patient mortality [94-99]. Intra-dialytic hypotension is ameliorated by higher dialysate [Na+], and is likely to be at least as deleterious (if not more so) as inter-dialytic hypertension.

Another concern with lower dialysate [Na+] is that it might influence serum [Na+]. Humans are considered to have an individual natremic set point, and most observational studies have not shown any cross-sectional correlation between dialysate and serum [Na+] [67,73,100-105]. However, pre-dialysis serum [Na+] did change in several small prospective clinical trials after changes to dialysate [Na+] [54,58,62,64,89,106,107], albeit often after a lag of several months possibly due to the large reservoirs of non-osmotic sodium in skin and bone [108-111]. There is a clear association between low serum [Na+] and patient mortality in patients with kidney disease, and an intervention that might potentially lower serum [Na+] warrants careful scrutiny [112-114].

A final concern is raised by clinical trials of dietary salt reduction in non-ESKD populations. Overall, there is higher mortality and morbidity in those participants who had the lowest salt intake, especially in the setting of generally low salt consumption [115-117]. A number of plausible biological mechanisms might be contributing to the poorer outcomes among those with low salt intake, involving several key metabolic and neurohormonal pathways (e.g. activation of sympathetic nervous and renin–angiotensin systems, increased in total and low-density lipoprotein cholesterol, reduction in peripheral insulin sensitivity etc.) [118-121].

**Figure 2:**
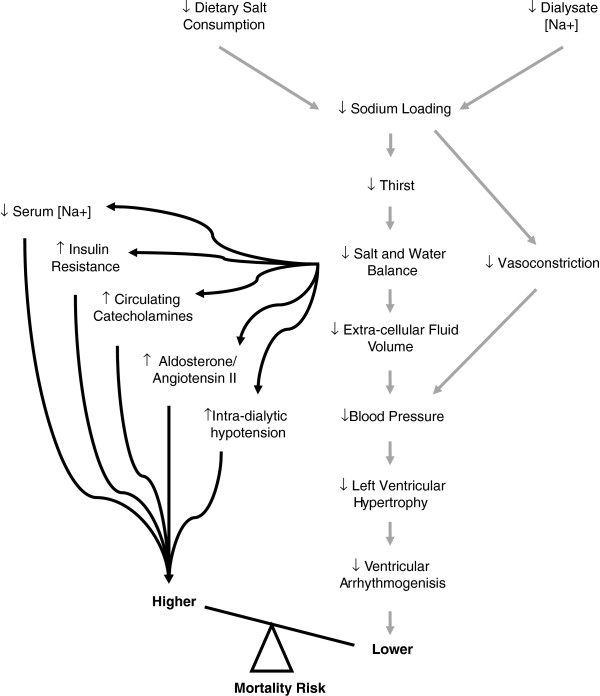
**Causal diagram relating low salt exposure during hemodialysis to cardiovascular mortality risk (reproduced with permission from Marshall and Dunlop **[41]**).**

Overall, there is clinical equipoise around lower dialysate [Na+] due to the multiple physiological consequences of reducing salt exposure in this population, and the uncertain net effect on CV outcomes as a result of these often competing and often conflicting physiological responses (Figure 2) [41]. The SOdium Lowering In Dialysate (SoLID) trial has been designed to answer the following clinical question: Does lower dialysate [Na+] improve CV mortality risk compared to conventional dialysate [Na+], in prevalent home HD patients who are exposed over a duration of a year? The research aims to address the clinical question through a randomised controlled trial of low versus standard dialysate [Na+], while observing the effect of allocation and exposure upon participant’s LV mass, an accepted surrogate outcome for CV mortality in end-stage kidney disease populations [12,22,23,36,122].

## Methods/design

### Study aim and hypothesis

The aim of this research is to examine the impact of low dialysate [Na+] on cardiovascular risk in patients on dialysis. Our primary hypothesis is that low dialysate [Na+] for one year among patients undergoing home HD will result in reduced LV mass. Our secondary hypothesis is that low dialysate [Na+] will also result in the following outcomes compared to conventional dialysate [Na+]: improved markers of thirst; better control of BP and markers of ECF volume; improved LV volumes and hemodynamics; decreased arterial stiffness; improved markers on long term CV mortality risk; non-inferior tolerance to dialysis; non-inferior plasma Na+ ionic activity and osmolarity; and non-inferior health-related quality of life.

### Study design and setting

**Figure 3:**
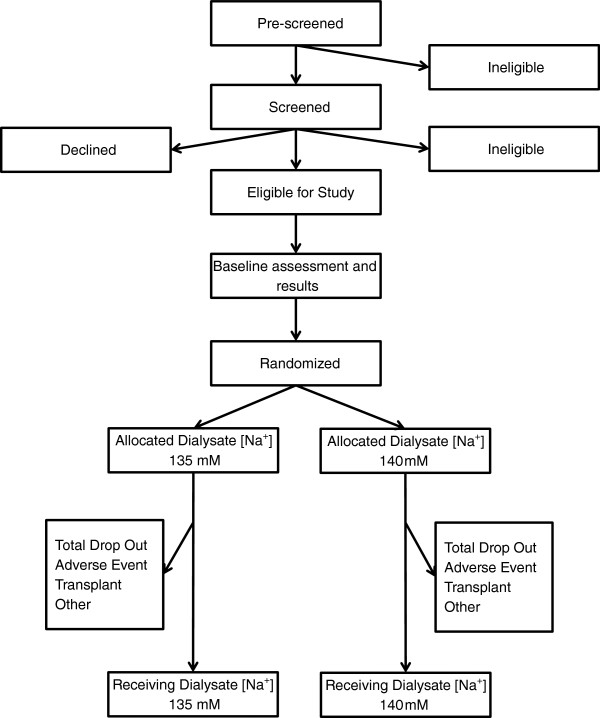
The SoLID trial participant flowchart.

The SoLID trial is a multi-centre prospective, randomised, single-blind (outcomes assessor), controlled, parallel assignment 3-year clinical trial (Figure 3). There will be accrual of participants over 24 months, and a follow up duration of 12 months. Participants will be randomly allocated to either low dialysate [Na+] of 135 mM or conventional dialysate [Na+] of 140 mM for 12 months duration, interventions that represent poles of customary practice with respect to dialysate [Na+] prescription in New Zealand. The trial will be conducted within 6 of the country’s 20 District Health Boards (DHBs), which are entities responsible for the provision of government-funded health and disability services in their geographical district. All of the DHBs in the SoLID trial provide renal services and have comprehensive home HD programmes: Counties Manukau, Waitemata, Auckland, Capital & Coast, and Canterbury. Participants will be either predominantly from urban settings (Counties Manukau, Waitemata and Auckland DHBs) or from a mixture of urban and rural ones (Capital & Coast, Canterbury, Waikato). For logistical reasons, no participant will be enrolled who lives more than 90–120 minutes from the hospital providing tertiary renal services.

### Ethical considerations

Ethical approval has been obtained through the National (New Zealand) Multi-region Ethics Committee and each institutional review board within participating DHBs.

### Target population and eligibility criteria

**Figure 4:**
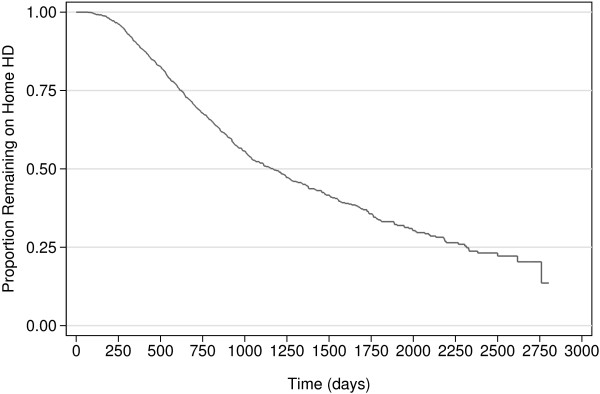
Kaplan-Meier estimates of non-death non-transplant censored home hemodialysis technique survival for the modern New Zealand population (2000–2010), from ANZDATA Registry; drop-out from home hemodialysis is 9.8% per year (15.3%, 53.6%, and 25.8% of drop-outs due to death, transplantation, and modality change respectively).

The target population will be patients with ESKD on home HD. The particular sampling frame for the SoLID trial is chosen because home HD patients have the large attributable risk of death from CV as opposed to other causes [3], and a less “frail” phenotype compared to those dialysing in facilities with less tendency to intra-dialytic hypotension [123,124]. They are also the least likely dialysis patients to suffer from other inter-current illnesses that might result in their death or drop out from the trial (Figure 4), and are probably more likely to be compliant with the study procedures.

Eligibility criteria will include incident or prevalent patients treated with maintenance home HD under the care of the 6 participating DHBs who are; aged 18 years or older; suitable for both low and standard dialysate [Na+] in the view of their treating physician; have pre-dialysis plasma [Na+] ≥ 135 mM; and are willing to participate and able to provide consent.

Exclusion criteria will include HD treatments at a frequency greater than 3.5 times per week; treatment with maintenance hemodiafiltration; life expectancy of less than 12 months; scheduled for live donor kidney transplantation within 12 months of entry to the study; considered by the treating nephrologist to have concomitant illnesses or conditions that limit or contraindicate study procedures and follow-up (e.g. frequent intra-dialytic hypotension requiring fluid resuscitation); considered by the treating nephrologist to have a high chance of non-adherence to study treatments and non-attendance for procedures and follow up; current enrolment in clinical studies involving anti-hypertensive medications, changes in HD operating parameters, or any other intervention that is likely to confound the outcome of the trial; currently using sodium profiling during haemodialysis treatments; documented infiltrative cardiomyopathies (amyloid, glycogen storage disease), hereditary cardiomyopathies (hypertrophic cardiomyopathy) or moderate to severe aortic valve disease (aortic stenosis, regurgitation); inability to provide consent or follow study instructions due to mental health illnesses or conditions.

### Recruitment of participants

The research team at each site will utilise a purpose built pre-screening database in order to identify potential participants eligible for inclusion in the trial. Potential participants will then be approached by either their dialysis nurse, doctor or the trial research co-ordinator and formally invited to consider participation. After an initial explanation, we will provide further written information and schedule a follow-up meeting. At that meeting, potential participants will be able to meet the site investigator with a language interpreter as necessary, and ask any questions that arise from information that has provided. If the potential participant is willing to participate, they will provide written consent. Whenever a signed consent form is received from a patient they will be formally screened to confirm their eligibility and then enrolled in the trial. Patients who are approached but decline to participate or consent but are not eligible when screened will be recorded in a screening log and their care will continue in the usual fashion.

### Randomisation

Once baseline assessments have been completed and 28 days have elapsed since enrolment onto the trial, randomisation will be performed using a phone based interactive voice response system (IVRS) from the National (Australia) Health Medical Research Council (NHMRC), Clinical Trials Centre (CTC) Sydney, Australia. Participants will be randomised in computer generated blocks of random size (undisclosed), blinded to investigators, and stratified by a) treating centre, and b) whether they receive conventional (≤18 hours/week) or extended-hour (>18 hours/week) HD.

### Blinding

Baseline data will be collected prior to randomisation so that trial investigators, research co-ordinators and trial participants will be blinded to allocation while the baseline data is being collected. However, blinding will be not be maintained once randomisation has occurred. Assessors for the primary outcome of the study will be blinded for the duration of the trial. Assessors for the secondary outcomes of the study will not be blinded.

### Interventions

It has been previously estimated that high sodium exposure during HD for most populations would be characterized by dialysate [Na+] of ~141 mM, and low sodium exposure by dialysate [Na+] of ~135 mM [101]. Increasingly, there are calls by opinion leaders for individualised dialysate [Na+] prescriptions as being the most physiological approach to manage sodium balance. Individualised prescriptions can be achieved through the automated application of sodium kinetic (or conductivity) models, or the manual application of simplified algorithms based on patient pre-dialysis [Na+] [102,125-131]. However, preliminary research by the SoLID research team has suggested that natremic adaptation may in fact occur over time in response to altered dialysate [Na+] [89]. Moreover, there is no evidence that the results achieved using individualised dialysate [Na+] prescriptions are better than those achieved with the simpler and less expensive intervention of a fixed lower dialysate [Na+] applied to everyone. Consequently, dialysate [Na+] in our study arms will be fixed rather than individualised for all participants. A 2010 poll of dialysate [Na+] by the SoLID research team has shown that the median setting for the New Zealand centres was 139 mM.

Low Dialysate [Na+]: This group will undergo home HD with dialysate [Na+] of 135 mM for a duration of one year, introduced gradually by decrements of 1 mM/week over a 4–8 week run-in period as necessary. BP will be optimized by changes to target weight and antihypertensive medications according to a standardized protocol.

Standard Dialysate [Na+]: This group will undergo home HD with dialysate [Na+] of 140 mM for a duration of one year, similarly introduced by changes of 1 mM/week over an appropriate run-in period. BP will be optimised in an identical manner as above.

In the event of titration failure, the dialysate [Na+] level reached while aiming for the target level will be retained for the remainder of the follow-up, although such cases will be classified as being protocol violations.

Apart from dialysate [Na+], HD operating parameters for all participants will be managed in usual fashion according to local treatment goals. Dietary salt intake will be managed in all participants according local clinical practice guidelines [132], and monitored at baseline, 6months and 12 months using 3-day food diaries and analyses with Foodworks Pro® 9.0 (Xyris Software, Brisbane, Australia). Urinary Na+excretion will be monitored in all participants at baseline, 6 months and one year follow-up using inter-dialytic urine collection.

### Research outcomes and endpoints

The schedule of SoLID trial investigations and visits are summarised in Table 1, and outcomes are described below.

**Table 1:** Schedule of participant investigations and visits

**Visit window (Days)**	**0 to28**	**28**	**28 to 42**	**42 up to 70**	**108 to 128**	**198 to 218**	**288 to 308**	**378 to 398**	^**+**^**7 up to **^**+**^**21**
**Visit name**	**BA/BR**		**T0**	**T1-T8**	**F3**	**F6**	**F9**	**F12/F12R**	**E1-E4**
**Study phase**	**Baseline assessment and results**	**Randomization**	**Titration (weekly)**		**Follow-up (3 monthly)**				**End (weekly)**
Randomization		X							
History	X					X		X	
Physical examination	X							X	
Non-interventional Titration			X						
Dialysate [Na^+^] Titration				X					
3day food diary	X					X		X	
Inter-dialytic urine for Na^+^ excretion	X					X		X	
Cardiac MRI	X							X	
ECF volume	X				X	X	X	X	
BP (intra-dialytic)	X			X	X	X	X	X	X
Ambulatory BP (inter-dialytic)	X							X	
Home BP (inter-dialytic)	X					X		X	
IDWG	X			X	X	X	X	X	
Antihypertensive medication history	X				X	X	X	X	
Dialysis Thirst Inventory	X					X		X	
Short Xerostomia Inventory	X					X		X	
Laboratory studies (NT-pro-BNP, hsCRP, urotensin II, plasma γNa / osmolality)	X				X	X	X	X	
Assessment of tolerance to HD	X			X	X	X	X	X	X
Arterial compliance (PWV)	X					X		X	
Arterial compliance (PWA)	X				X	X	X	X	
Quality of life (KDQOL)	X							X	
Quality of life (EQ-5D)	X							X	

#### Primary outcome – LV mass index

The primary outcome measure of the SoLID trial is LV mass index (LVMI), and the primary endpoint is LVMI at 12months. LVMI is also measured at baseline. LVMI will be measured using cardiac magnetic resonance imaging (MRI) imaging performed prior to HD treatments after a “long break” (the longest HD-free interval in any rolling schedule) or mid-week for participants who have fixed inter-dialytic intervals. All cardiac MRI scans will be performed at the local sites using a standardised protocol. Assessment of LV function will be performed using trueFISP cine imaging (6–7 short axis and 3 LV long axis with 20–30 cardiac phases depending on heart rate). Analysis of the images will be performed at a core laboratory at the Auckland MRI Research Group, University of Auckland, New Zealand. Each patient will have a four-dimensional mathematical model of the left ventricle created using guidepoint fitting. In all cases, volume, mass and wall thickness will be measured directly from the moving 3D curved surfaces which track the motion of the endo- and epicardium. This method has been validated for global parameters such as LV mass, end-diastolic volume, end-systolic volume, stroke volume and ejection fraction using global gold standard models [133]. All data will be analysed in duplicate by two independent and blinded analysts and the results reconciled in accordance with standard operating procedures of the group. Analysts will be monitored weekly for drift.

#### Secondary outcomes

##### LV volumes

LV volumes will be measured by cardiac MRI at baseline, and the endpoint will consist of follow-up measurements at 12 months. Measurements will be made used the same methodology used for LV mass index.

##### LV hemodynamics

LV hemodynamics will be as assessed by NT-pro-BNP (N-terminal pro brain natriuretic peptide) [134-140] and Urotensin II levels [141-145]. Measurements will be made at baseline, and the endpoints will consist of measurements at 3, 6, 9 and 12 months. All blood samples will be taken immediately prior to HD treatment following a “long break” or mid-week for participants who have fixed inter-dialytic intervals. All measurements will be made by the Christ church Cardiac Endocrine group in Christ church, New Zealand using the Elecsys® ProBNP assay (Roche Diagnostics Corporation, Indianapolis, IN, USA) and an in-house radioimmune assay for Urotensin II [146].

##### Extracellular fluid volume

ECF volume will be assessed by bioimpedance spectroscopy [147-149]. Measurements will be made at baseline, and the endpoints will consist of measurements at 3, 6, 9 and 12 months. All assessments will be performed immediately prior to HD treatments after a “long break” or mid-week for participants who have fixed inter-dialytic intervals. All measurements will be made using the Fresenius BCM monitor® (Fresenius Medical Care Australia Pty Ltd (New Zealand Branch), Auckland, New Zealand).

##### Blood pressure

BP will be assessed in the following ways;

a. Intra-dialytic BP (including pre- and post-dialysis BP). Measurements will be made at baseline, and the endpoints will consist of measurements at 3, 6, 9 and 12 months [150,151]. An additional endpoint will consist in the intra-dialytic blood pressure time-averaged over the individual follow-up period, using all available measurements accepting that there may be differing numbers of measurements per participant and time point.

b. Inter-dialytic BP assessed by the gold standard of ambulatory monitoring [150,152-155]. Measurement will be made at baseline, and the endpoint will consist of measurement at 12 months. All assessments will be performed according to the ‘Practice Guidelines of the European Society of Hypertension’ for clinic, ambulatory and self BP measurement [156]. Systolic and diastolic BP measurements will be made at 30 minute measurement intervals for 44 hours during a “long break” or mid-week for participants who have fixed inter-dialytic intervals. All measurements will be made using Oscar 2 ambulatory BP monitors (Suntech Medical Instruments, Raleigh, NC, USA). Measurements will be made on the non-access arm, and the measurement arm will be kept consistent during the study. Ambulatory BP monitoring will be considered adequate if at least two-thirds of the measurements taken over the 44-hour period are satisfactory [156].

c. Inter-dialytic BP determined as the weekly average of home blood pressure readings, an independently validated approach [150,157,158]. Measurements will be made at baseline, and the endpoints will consist of measurements at 6 and 12 months. Measurements will made using the Omron HEM-791IT Blood Pressure Monitor (Omron Healthcare Inc, Lake Forest, IL, USA). Readings will be made 3 times per day (on waking up, between noon and 1900, and before bed) during the 7days immediately prior to 3, 6, 9 and 12 months [150,157,158].

d. The number and dose of anti-hypertensive medications (expressed as the aggregated% of maximum recommended daily dose) [159]. Measurements will be made at baseline, and the endpoints will consist of measurements at 3, 6, 9 and 12 months.

##### Inter-dialytic weight gain (IDWG)

IDWG will be calculated by the difference between pre- and- post dialysis weight. Measurements will be made at baseline, and the endpoints will consist of measurements at 3, 6, 9 and 12 months. An additional endpoint will consist in the inter-dialytic weight gain time-averaged over the individual follow-up.

##### Thirst and xerostomia

Thirst and xerostomia will be assessed by visual analogue scale using standardised validated inventories [160-163]. Measurements will be made at baseline, and the endpoints will consist of measurements at 3, 6, 9 and 12 months.

##### High sensitivity C-reactive protein (hsCRP)

Long term CV mortality risk will be assessed by hsCRP [164]. Measurements will be made at baseline, and the endpoints will consist of measurements at 3, 6, 9 and 12 months. All blood samples will be taken immediately prior to HD treatment following a “long break” or mid-week for participants who have fixed inter-dialytic intervals. All measurements will be made using the Abbott Architect® Analyser and CRP Vario® latex immunoassay (Abbott Park, IL, U.S.A).

##### Arterial compliance

Arterial stiffness will be assessed by carotid-femoral Pulse Wave Velocity (PWV) and by radial Pulse Wave Analysis (PWA, deriving both central pulse pressure and the augmentation of the central pulse waveform at the radial artery). The methodology represents the current gold standard for non-invasive measurement of aortic arterial stiffness [165]. Pulse Wave Velocity measurements will be made at baseline, and the endpoints will consist of measurements at 3, 6, 9 and 12 months. Pulse Wave Analysis measurements will be made at baseline, and the endpoints will consist of measurements at 6 and 12 months. Assessments will be performed immediately prior to HD treatments after a “long break” or mid-week for participants who have fixed inter-dialytic intervals. Measurements will be made using the SphygmoCor® device (AtCor Medical, West Ryde, Australia) which employs applanation tonometry to measure the shape and velocity of the pulse wave, with good repeatability and reproducibility. Both PWV and PWA measurements will be measured in duplicate. If the difference between the two PWV measurements is > 10%, a third measurement will be performed and the average the two most similar measurements used. For quality control, the Sphygmocor calculates the standard deviation (SD) of the pulse transit time (PTT) over the 10 second waveform capture period. A carotid-femoral PTT SD of < 20% in dialysis patients is considered by the manufacturers to indicate a good quality measurement for PWV and will be the accepted maximum carotid-femoral PTT SD for measurements in this trial.

##### Pre-dialysis plasma sodium ionic activity (γNa)

Pre-dialysis plasma γNa and osmolality will be assessed at baseline, and the endpoints will consist of measurements at 3, 6, 9 and 12 months. All blood samples will be taken immediately prior to HD treatment following a “long break” or mid-week for participants who have fixed inter-dialytic intervals. Plasma γNa measurements will be made by direct inometry using an ABL800 or ABL83 blood gas analyser (Radiometer, Copenhagen, Denmark) and corrected by a factor of 0.967 to account for the Donan Effect from negatively charged plasma proteins [166]. Plasma osmolality will be analysed by freezing point depression using the Advanced® Model 3320 Micro-Osmometer (Advanced Instruments, Norwood, MA, USA).

##### Health related quality of life

Consistent with previous recommendations [167-169], health related quality of life (HRQoL) will be assessed using a spectrum of validated instruments. Measurement will be made at baseline, and the endpoint will consist of measurement at 12 months. Measurements will be made using the Kidney Disease Quality of Life (KDQOL) [170-172] and the preference based EuroQol EQ-5D questionnaires [167,173].

#### Tolerability outcome

##### Tolerance to dialysis

Tolerance to dialysis will be assessed by the frequency of intra-dialytic hypotension episodes in the two weeks prior to the assessment time point. Hypotension episodes will be identified according to the NKF-K/DOQI definition (a decrease in systolic BP by ≥20 mMHg or a decrease in mean arterial pressure by ≥10 mMHg associated with one or more of the following symptoms: abdominal discomfort, nausea, vomiting, muscle cramps, restlessness, dizziness, fainting, anxiety and the requirement for fluid boluses) [164]. Measurements will be made at baseline, and the endpoints will consist of measurements at 3, 6, 9 and 12 months. An additional tolerance endpoint will consist in the time-averaged frequency of hypotension episodes over the individual follow-up period.

### Monitoring for adverse events

A formal Data Monitoring Committee (DMC) constituted by the New Zealand Health Research Council Data Monitoring Core Committee will monitor safety and trial conduct according to the terms of its charter. An independent study statistician and data manager will generate both the open and closed Reports for the DMC, and have no connection to the clinical aspects of the trial. Because of power considerations and the fact that safety of low dialysate [Na+] was demonstrated in the pilot study, no interim analyses are planned and no stopping rule based on statistical significance of efficacy data, frequentist or Bayesian, has been set. However, safety reports will be made and reviewed by the DMC on a 6 monthly basis.

### Power calculation

Power calculations are based on the primary outcome measure of LVMI. The SoLID trial assumes a baseline mean (standard deviation, SD) LVMI of 110 (40) g/m2, based on published data in HD populations using cardiac MRI [33,174,175]. The trial assumes a 12 month follow-up LVMI of 95 (35) g/m2 in the low dialysate [Na+] group based on the change in LVMI observed over 6 months in clinical trials of frequent or nocturnal HD [27,34,35]. Correlation between baseline and 12-moth follow-up measurements of LVMI in the SoLID trial is assumed to be 0.75 based on private communication from the Jardine group (private communication P Mark 8/2/2011): in a cohort of 59 patients of their patients with repeated measures of LVMI at least 6 months apart (using cardiac MRI), correlation was 0.87 (p < 0.001) with normally distributed data [33,174,175]. Modelling these data using repeated-measures analysis of covariance (ANCOVA), and allowing for 25% for drop outs, 59 participants will be enrolled in each arm (power 0.8, alpha 0.05). The SoLID trial will therefore enrol 118 trial participants over the 6 participating sites in NZ.

### Analysis populations

For analysis of data, we define Intention to Treat (ITT) and Per Protocol (PP) populations. The ITT population consists of all randomised participants who have at least one baseline measurement, and is the primary population of interest. All randomised participants will be analysed in the group they were allocated to, even if they do not receive the allocated treatment, do not commence treatment, change dialysis modality, are lost to follow-up, or die thereby preserving the intention-to-treat framework. In particular, titration failures will remain within the ITT population as participants.

The PP population consists of participants that fulfil criteria for the ITT population, have complete primary endpoint measurements and do not present any major protocol violations during the study. The following describes the major protocol deviations that will exclude patients from the PP population (minor deviations will not do so): eligibility violation; absence of any efficacy data, titration failure; other major violations will be identified by the DMC of the trial during the study and/or during the data review process. The list of all protocol deviations will be reviewed by the DMC who will determine the degree of the violation (i.e. major versus minor). All protocol deviations considered as minor will not lead to excluding patients from the PP population for analysis.

### Statistical analysis

Primary and secondary subgroup and non-subgroup analyses are provided in Table 2. For statistical analyses, we define predictors, related to outcome and unrelated to the allocation; potential confounders, related to outcome and imbalanced by chance across the treatment arms; and potential effect modifiers, that may moderate the treatment arm effect. The former two are hereafter identified as potential covariates.

**Table 2:** Primary and secondary analyses

**Analyses**	**Population**	**Subgroup**	**Endpoints**	**Framework**
Primary	ITT	None	Primary	Univariate
Secondary	PP	None	Primary	Univariate
	ITT	None	Secondary	Univariate
	ITT	None	Secondary	Multivariate
	ITT	Baseline LVMI subgroups (observed median LVMI as the level boundary)	Primary	Univariate
	ITT	Baseline LVMI subgroups (observed median LVMI as the level boundary)	Time-averaged blood pressure over months 3, 6, 9 and 12 (intra-dialytic, inter-dialytic), % maximum recommended daily dose of antihypertensives	Multivariate, accounting for subgroup effect and treatment-subgroup interaction, FDR control to account for multiplicity
	ITT	Baseline intra-dialytic and inter-dialytic blood pressure subgroups (observed mean blood pressure as the level boundary)	Primary	Univariate, accounting for subgroup effect and treatment-subgroup interaction, and three-way interaction
	ITT	Baseline intra-dialytic and inter-dialytic blood pressure subgroups (observed mean blood pressure as the level boundary)	Time-averaged blood pressure over months 3, 6, 9 and 12 (intra-dialytic, inter-dialytic), % max recommended daily dose of antihypertensives	Univariate, accounting for subgroup effect and treatment-subgroup interaction, and three-way interaction, FDR control to account for multiplicity
	ITT	Baseline pre-dialysis plasma Na+ ionic activity subgroups (observed median plasma Na+ ionic activity as the level boundary)	Primary	Univariate, accounting for subgroup effect and treatment-subgroup interaction
	ITT	Baseline pre-dialysis plasma Na+ iIonic activity subgroups (observed median plasma Na+ ionic activity as the level boundary)	Time-averaged blood pressure over months 3, 6, 9 and 12 (intra-dialytic, inter-dialytic), % maximum recommended daily dose of antihypertensives	Univariate, accounting for subgroup effect and treatment-subgroup interaction, and three-way interaction, FDR control to account for multiplicity
Tolerability	ITT	None	Hypotension event counts	Multivariate, mixed effects, allowing for treatment time interaction

Abbreviations: *PP* per protocol, *ITT* intention to treat, *LVMI* left ventricular mass index, *FDR* False Discovery Rate.

All tests of significance of hypotheses concerning treatment effect parameters will be carried out using a level of significance of 5% and two-sided alternatives. The significance threshold of potential covariates will be set at 10%, to promote unbiased and conservative inference. All estimates will be produced as point estimates and as 95% confidence intervals. Unless otherwise noted, model selection when required will be performed using backward selection from the largest model dictated by the situation. Per comparison error rate (PCER) control will be used in all analyses, with the exception of some subgroup analyses where False Discovery Rate (FDR) control will be implemented.

### Primary outcome

For LVMI, we will undertake univariate (single outcome) endpoint analysis. Score residuals will be checked for approximate normality and an appropriate normalising transformation applied to the endpoint data if appropriate and possible. We will use analysis of covariance (ANCOVA) to estimate the treatment effect, adjusting for baseline and potentially adjusting for covariates, if applicable. The resulting treatment contrast will be reported as a point estimate and as a 95% confidence interval.

### Secondary outcomes

For all secondary outcomes other than health-related quality of life, we will undertake multivariate (repeated outcomes) endpoint analysis. For health-related quality of life, we will use univariate analyses in a manner similar to that presented for the primary outcome. Otherwise, analyses will fit all outcomes as repeated measures over the 2–4 assessment time points to an appropriate regression model using generalised estimating equations (GEEs). Changes from baseline will be associated with the assessment time (from baseline), the treatment arm, and the interaction of the two, adjusting for baseline and potentially for other confounding covariates, if applicable, similarly to the procedure outlined above for the primary outcome. The procedure will account for clustering on subjects; independent, exchangeable and autoregressive working correlation structures will be used and the best option in terms of Quasilikelihood Information Criterion retained for the final analyses [176]. Time-averaged analyses will be implemented by appropriately weighting each observation, accounting for any missing time point, so as to produce inference on the estimated average value over the available individual follow-up period. All results will be estimates of treatment effect and treatment-time interaction contrasts. Results will be reported as the point estimates and 95% confidence intervals for these quantities.

### Contingency for non-normality

Equivalent analyses after a normalising data transformation will be carried out if non-normality of outcomes is evinced. The choice of transformation will be guided by the stabilisation of variance. Notable departures from normality of residuals after regression of the transformed data, as evinced by visual assessments and formal tests of normality of residuals, will result in an alternative approach. When a transformation is applied, location estimates and confidence intervals will be transformed back to the original scale, with first-degree bias correction.

### Missing data

Every effort will be made to minimise missing data. Missing outcome data will cause the patient/time point instance to be removed from the analysis. In the case of time-averaged endpoints, non-missing data will be appropriately reweighted. Joint modelling of missingness and the primary outcome will be carried out, for sensitivity assessment, if missingness exceeds 10% or is significantly different between the intervention arms at the 5% level.

### Subgroup analyses

Subgroup analyses will be performed for the primary outcome and time-averaged BP (intra-dialytic, inter-dialytic, percentage maximum recommended daily dose of antihypertensives) according to the baseline severity of LV hypertrophy, baseline severity of hypertension, and baseline pre-dialysis plasma γNa. Subgroup analyses will be carried on using interaction of the groups thus defined and the treatment arms.

### Tolerability analysis

Tolerability of dialysis will be compared between the two arms by assessing the frequency of intra-dialytic hypotension episodes using negative binomial regression of the number of events as dependent variable and the logarithm of the duration of the period covered (normally two weeks, prior to assessment). To identify any effect of prolonged exposure to the treatment, the dependent variable will be similarly modelled as a repeated measure on the time of assessment, the treatment arm and their interaction. To maintain interpretability at the patient level, conditional (mixed effects) rather than marginal (GEE) methodology will be used to estimate tolerability trends. Normally distributed random effects will account for the baseline dialysis tolerance in each participant. The treatment and treatment interaction contrasts from the tolerability analyses will be reported as point estimates and 95% confidence intervals.

## Discussion

The SoLID trial will be the first randomised controlled trial to investigate the effect lower dialysate [Na+] upon LV structure and function. The outcomes of this research will provide compelling evidence about the efficacy of lower dialysate [Na+] to improve CV outcomes in hemodialysis populations. As importantly, the SoLID trial will provide novel data with respect to important patient centred outcomes, and evaluate the effect of lower dialysate [Na+] on thirst, xerostomia, HRQoL and intra-dialytic hypotension. If the benefit of lower dialysate [Na+] is confirmed, other benefits might also flow on from reduced CV morbidity and mortality, including improvements in general health status, and conceivably fewer hospitalisations and improvements in social/employment rehabilitation. Logistically, lower dialysate [Na+] is a simple and cost-free intervention, and widespread implementation would be easy for home HD patients and selected facility HD patients.

The comprehensive range of secondary outcomes and the use of gold standard measurements further strengthen the trial. These data allows for their simultaneous evaluation as intermediary variables on the causall pathway to LV hypertrophy. Such data have not been previously reported in the setting of clinical trial, where fundamental changes are made to conditions with simultaneous measurement of downstream physiological parameters. Such data will improve understanding of the patho-biology and causall mechanisms of LV hypertrophy, and in particular the contributions of uncontrolled hypertension and ECF overload. Data from the SoLID trial will allow the development of a hierarchy of importance for the various factors that increase LV mass. There is a strong likelihood of novel relationships and hypotheses emerging, in turn leading to further research in both bench and clinical settings.

In terms of internal validity, the strengths of the SoLID trial are that it is prospective and randomised, with robust allocation concealment and analysis of cardiac MRI data in duplicate by two blinded independent analysts who will remain unaware of the allocation of participants for the duration of the trial. A limitation of the trial pertains to secondary outcomes. All baseline measurements of secondary outcomes will be made prior to randomisation. However, once randomisation has occurred, participants and assessors for the follow-up measurements will not be blinded to treatment. For instance, bioimpedance and pulse wave procedures and measurements will be performed by research associates who will be potentially aware of the allocation of participants. This may bias participants’ and research associates’ perception of tolerance to the intervention, and their reporting of subjective scoring surveys such as thirst and xerostomia inventories and HRQoL questionnaires. The impact of any actual bias is likely to be mitigated through completion is baseline measurements prior to randomisation, but certainly not abrogated.

Of note, the SoLID Trial excludes those who are on dialysis more than 3.5 times per week to avoid confounding the effect of dialysate [Na+] on LV mass by frequent or nocturnal dialysis. The study will not exclude those whose HD treatments are unconventionally long (~8 hours per treatment) so long as they are undergoing HD no more than 3.5 times per week. This is based on the high reported prevalence of LV hypertrophy in populations treated with this manner (notwithstanding the overestimation of LV mass by echocardiography that biases these studies [175]): 87% of patients in Christchurch NZ [124], 76% of those in Manchester UK [177], and in more than 80% of those in Tassin France [178]. LV hypertrophy is still present in these populations on long-hour dialysis as a result of BP variability, neurohormonal factors and residual positive sodium balance, all of which are potentially modifiable with lower dialysate [Na+].

In terms of external validity, the major limitation of the SoLID trial is the research setting of home HD. Participants are likely to have less medical co-morbidity than facility HD patients, a factor that was a prime consideration in the development of the sampling frame for the SoLID trial. Such patients are less prone to intra-dialytic hypotension, which minimizes the chances of participant dropout from intolerance to the lower dialysate [Na+] in the trial. As a result, the SoLID trial may not be immediately applicable to dialysis patients with a high burden on medical co-morbidity who are more prone to intra-dialytic hypotension. Notwithstanding, the SoLID trial may provide valuable proof-of-concept data for future research in this patient population, depending on the clinical characteristics of patients eventually recruited to the trial.

As with any clinical trial, recruitment will be a key element. Feasibility analysis of the participating 6 centres has indicated a sufficiently large pool of potential participants assuming a 33% conversion rate. A pilot study previously undertaken by the SoLID research group showed good tolerance to lower dialysate with minimal adverse events, and we do not anticipate excess loss of participants due to intolerance of the trial intervention [89]. The 25% dropout rate we modelled in power calculations accounts for technique failure in the modern NZ home HD population. A potential risk to recruitment lies with the collection of so many secondary endpoints and their need to occur at specific times on the participants’ dialysis schedules, which may make the trial unattractive to some patients. Slow recruitment will be managed by the expansion of the SoLID trial to include a 7^th^ site in NZ or Australia, as required.

In conclusion, the SoLID trial will provide compelling evidence about the use of lower dialysate [Na+] to improve CV outcomes, and potentially improve understanding of the biological mechanisms underlying the development and persistence of LV hypertrophy. If the benefit of lower dialysate [Na+] is confirmed, the SoLID Trial will contribute a cost-free economically sustainable improvement to dialysis practice. This, and the immediately availability of the intervention to HD patients, will allow for optimal and maximal translation into clinical effectiveness and benefit.

## Abbreviations

HD: Hemodialysis; ESKD: End-stage kidney disease; ANZDATA: Australian and New Zealand dialysis and transplant registry; SCD: Sudden cardiac death; LV: Left ventricular; LVH: Left ventricular hypertrophy; ECF: Extra-cellular fluid; BP: Blood pressure; IDWG: Intra-dialytic weight gain; LVMI: Left ventricular mass index; CAMRI: Centre for advanced magnetic resonance imaging; DMC: Data monitoring committee; CV: Cardiovascular; CKD: Chronic kidney disease; LDL: Low density lipoprotein; IVRS: Interactive voice response system; NHMRC: National (Australia) health medical research Council; CTC: MRI: Magnetic resonance imaging; NT-pro-BNP: N Terminal pro Brain Natretic Peptide; BCM: Body composition monitor; hsCRP: high sensitivity C-Reactive Protein; PWV: Pulse wave velocity; PWA: Pulse wave analysis; SD: Standard deviation; PTT: Pulse transit time; ϒNa: Ionic sodium; NKF-K/DOQI: National kidney foundation kidney disease outcomes quality initiative; HRQoL: Health related quality of life; SOLID: Sodium lowering in dialysis; ANCOVA: Analysis of Co-variance; ITT: Intention to treat; PP: Per protocol; PCER: Per comparison error rate; GEE: Generalised estimating equation; FDR: False discovery rate.

## Competing interests

The authors declare that they have no competing interests.

## Authors’ contributions

JLD participated in the trial design, and drafted the manuscript. ACV participated in the trial design, developed the statistical plan, and helped to draft the manuscript. MRM conceived and developed the trial, and participated in the statistical plan and helped to draft the manuscript. RSG, IAH, and DORM participated in design and development of the trial, and helped to draft the manuscript. JRdZ and PJM participated in the trial design and coordination, and helped to draft the manuscript. CJH, KSR, and DJS participated in the trial coordination and implementation, and helped to draft the manuscript. All authors read and approved the final manuscript.

## Authors’ information

JLD is a Senior Lecturer at the Auckland Clinical School, Faculty of Medical and Health Sciences, University of Auckland, Private Bag 93311, Otahuhu, Auckland 1640, New Zealand, and a specialist nephrologist within the Department of Renal Medicine, Middlemore Hospital, Counties Manukau District Health Board, Private Bag 93311, Otahuhu, Auckland 1640, New Zealand. ACV is a statistical mathematician within the Faculty of Health and Environmental Sciences, Auckland University of Technology, North Shore Campus, Private Bag 92006, Auckland 1142, New Zealand. JRdZ is a specialist nephrologist within the Renal Service, North Shore Hospital, Waitemata District Health Board, Private Bag 93503, Takapuna, Auckland 0740, New Zealand. RSG is a specialist cardiologist within the Department of Cardiology, Middlemore Hospital, Counties Manukau District Health Board, Private Bag 93311, Otahuhu, Auckland 1640, New Zealand. IAH and DJS are specialist nephrologists within the Department of Renal Medicine, Auckland City Hospital, Auckland District Health Board, Private Bag 92024, Auckland, Auckland 0740, New Zealand. CJH is a specialist nephrologist within the Department of Renal Medicine, Middlemore Hospital, Counties Manukau District Health Board, Private Bag 93311, Otahuhu, Auckland 1640, New Zealand. PJM is a specialist nephrologist within the Department of Nephrology, Wellington Hospital, Capital & Coast District Health Board, Private Bag 7902, Wellington South, New Zealand. DORM is a specialist nephrologist within the Department of Nephrology, Christchurch Hospital, Canterbury District Health Board, Private Bag 4710, Christchurch, New Zealand. KSR is a specialist nephrologist within the Department of Renal Medicine, Waikato Hospital, Waikato District Health Board, Private Bag 3200, Hamilton 3240, New Zealand. MRM is an honorary Associate Professor at the Auckland Clinical School, Faculty of Medical and Health Sciences, University of Auckland, Private Bag 93311, Otahuhu, Auckland 1640, New Zealand, and the Clinical Director of the Department of Renal Medicine, Middlemore Hospital, Counties Manukau District Health Board, Private Bag 93311, Otahuhu, Auckland 1640, New Zealand.

## Pre-publication history

The pre-publication history for this paper can be accessed here:

http://www.biomedcentral.com/1471-2369/14/149/prepub
